# Mitochondrial Contributions to Hematopoietic Stem Cell Aging

**DOI:** 10.3390/ijms222011117

**Published:** 2021-10-15

**Authors:** Claudia Morganti, Keisuke Ito

**Affiliations:** 1 Ruth L. and David S. Gottesman Institute for Stem Cell and Regenerative Medicine Research, Albert Einstein College of Medicine, Bronx, NY 10461, USA; claudia.morganti@einsteinmed.org; 2 Departments of Cell Biology and Stem Cell Institute, Albert Einstein College of Medicine, Bronx, NY 10461, USA; 3 Department of Medicine, Montefiore Medical Center, Albert Einstein College of Medicine, Bronx, NY 10461, USA

**Keywords:** hematopoiesis, hematopoietic stem cell, aging, mitochondrial metabolism, stem cell exhaustion, ROS, inflammation

## Abstract

Mitochondrial dysfunction and stem cell exhaustion are two hallmarks of aging. In the hematopoietic system, aging is linked to imbalanced immune response and reduced regenerative capacity in hematopoietic stem cells (HSCs), as well as an increased predisposition to a spectrum of diseases, including myelodysplastic syndrome and acute myeloid leukemia. Myeloid-biased differentiation and loss of polarity are distinct features of aged HSCs, which generally exhibit enhanced mitochondrial oxidative phosphorylation and increased production of reactive oxygen species (ROS), suggesting a direct role for mitochondria in the degenerative process. Here, we provide an overview of current knowledge of the mitochondrial mechanisms that contribute to age-related phenotypes in HSCs. These include mitochondrial ROS production, alteration/activation of mitochondrial metabolism, the quality control pathway of mitochondria, and inflammation. Greater understanding of the key machineries of HSC aging will allow us to identify new therapeutic targets for preventing, delaying, or even reversing aspects of this process.

## 1. Introduction

Aging is a time-dependent degenerative process that affects all living organisms. Since the aging population is inexorably growing, there is an imminent need to develop new therapeutic strategies for ameliorating the age-related changes and/or disorders, and first among these is hematopoietic aging. The milestone review paper of Reference [1] has categorized the cellular and molecular hallmarks of this type of aging, which include both mitochondrial dysfunction and stem cell exhaustion, the two main topics of this review.

Mitochondria were described as contributing to aging and degeneration as early as already in the 1920s, when the “rate of living hypothesis” proposed that metabolic rates inversely correlate with organismal lifespan [2]. Many researchers have since described the links between mitochondrial biology and aging [3,4,5,6]. Stem cell exhaustion refers to an impaired functionality of stem cells, which cannot maintain in the tissue in which they reside. In particular, aging of the hematopoietic system displays decreased immune response, declining immuno-competence, increased autoimmunity, diminished stress response, late-onset anemia, reduced regenerative capacity, and increased predisposition to a spectrum of diseases, including myelodysplastic syndrome (MDS) and acute myeloid leukemia (AML) [7,8,9,10].

**Figure 1:**
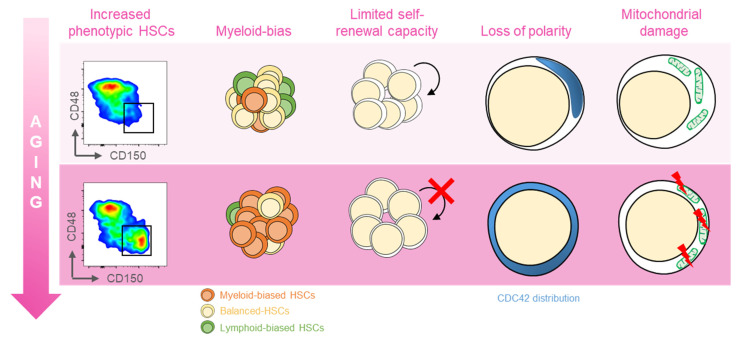
Features of aged HSCs. Upon aging, hematopoietic stem cells (HSCs) acquire phenotypical and functional peculiar properties. Flow cytometry analysis of the murine bone marrow shows an increase in the phenotypic HSCs (defined as c-Kit^+^Sca-1^+^Lin^−^CD135^−^CD48^−^CD150^+^) in old (18 months old, bottom panel) mice (far left). The HSC pool includes balanced HSC, which in equal proportion differentiate in myeloid and lymphoid lineage (yellow, left); with age, the myeloid-biased differentiation prevails at the expense of lymphoid cells (left). The self-renewal capacity typical of HSCs is reduced upon aging (middle). Cytoskeletal polarity detected by CDC42 localization is lost in old HSCs where CDC42 expression is homogeneously distributed (right). Aged HSCs display the mitochondrial damage with the altered metabolism (far right).

Several distinct features characterize aged hematopoietic stem cells (HSCs) (Figure 1) [11]. For example, it has been shown that phenotypic HSCs in the bone marrow increase in frequency with age, while losing their functionality. Multiple studies have demonstrated a differentiation bias toward the myeloid lineage; the aged murine hematopoietic system is impaired in supporting leukocyte numbers, erythropoiesis, and both B- and T-lymphoid cells in peripheral blood, while the numbers of myeloid cells are increased [12]. These myeloid-biased HSCs express high levels of CD150 (signaling lymphocyte activation molecule, or SLAMF1) and CD41 (integrin alpha 2, or Itga2b) proteins, which have been used to identify the HSC clonal subtypes responsible for hematopoietic aging [13,14]. As expected, stemness decreases in aged HSCs, which show reduced in vivo repopulation capacity, as determined by serial transplantation assays [15], along with a 3-fold lower efficiency in bone marrow homing after transplantation (Figure 1) [16]. 

Another key feature of aged HSCs is the loss of cell polarity. Young and aged long-term (LT) HSCs exhibit different distribution patterns of CDC42, Tubulin, and AcH4K16, which is believed to be caused by the elevated activity of CDC42, and is linked to age-associated changes in self-renewal and differentiation capacity [12]. Additionally, HSC aging results from cumulative cellular and genomic damage, which leads to permanent cell-cycle arrest, apoptosis, or senescence [15,17,18]. 

Although historically DNA damage was thought to be the main cause of HSC aging, many new findings have defined an increasing number of biological processes that intrinsically change with age in HSCs. These include epigenetics, chromatin architecture, autophagy, proteostasis, and metabolic changes [19].

**Figure 2:**
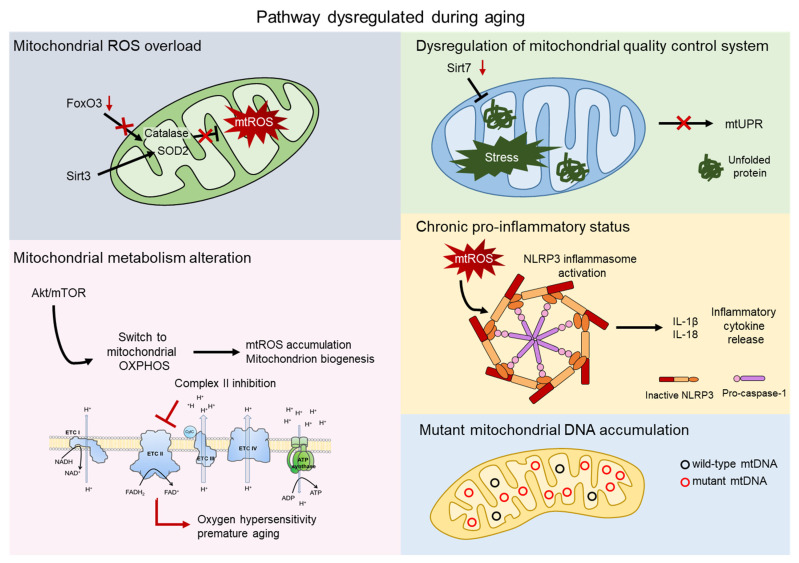
Mitochondrial contribution to aging. Aging process shows dysregulation of several pathway involving the direct contribution of mitochondria. Mitochondrial reactive oxygen species (mtROS) accumulate after the impairment of the major antioxidant defense systems, consist of Superoxide dismutase 2 (SOD2) and Catalase, caused by the aged-related loss of FOXO3 and SIRT3 (grey). AKT/mTOR signaling is a key regulator of mitochondrial metabolism and, upon aging, its imbalance causes mtROS accumulation, mitochondrial biogenesis, and an altered metabolism. Inhibition of complex II of the electron transport chain (ETC) leads to oxygen hypersensitivity and premature aging of HSCs (pink). The mitochondrial unfolded protein response system (mtUPR) is affected by the aged-related loss of *SIRT7*, leading to accumulation of unfolded protein and mitochondrial stress (green). Accumulation of mtROS activates the NOD-, LRR-, and pyrin domain-containing 3 (NLRP3) inflammasome, which triggers the release of inflammatory cytokines, such as IL-1β and IL-18 (orange). Mitochondrial dysfunctions include accumulation of mtDNA mutations in aged HSCs (blue).

Since aged HSCs generally exhibit enhanced mitochondrial oxidative phosphorylation (OXPHOS) and increased production of reactive oxygen species (ROS), it has often been proposed that mitochondria play a direct role in compromising HSC functions [20,21]. Here, we explore multiple aspects of the impact of the mitochondria on HSC aging. Several mechanisms have been reported to contribute to aging, such as mitochondrial ROS production, alteration in mitochondrial metabolism, and mitochondrial quality control pathways (Figure 2).

## 2. Mitochondrial ROS

Since the frequency of HSCs with low levels of ROS decreases with age, ROS generation/accumulation can be considered a distinctive characteristic of aging [22]. Proper levels of ROS are important mediators of various signal transduction pathways. However, increased levels of ROS affect HSCs’s lifespan [20], self-renewal [23,24], and differentiation [25,26]. ROS contribute to HSC aging and senescence, and excessive ROS generation induces apoptotic cell death in HSCs [20,23,27,28]. Increase in ROS levels in adult HSC have similarities with the aging phenotypes, such as myeloid lineage skewing and defective long-term repopulation activity [29,30]. On the other hand, very low levels of intracellular ROS in HSCs are essential to maintaining HSCs quiescence [22]. Mitochondria produce around 90% of cellular ROS, and the impairment of mitochondrial function, for example, by the loss of the Polycomb repressor *BMI1*, causes a major increase in intracellular ROS [31]. Aging of mitochondria leads to an overload of ROS, which further damage the mitochondria, resulting in perpetual cell cycling [32]. Evidence for this has come from studies of the effects of FOXO transcription factors, key players in the oxidative stress response, on HSC fitness. Genetic variation within the *FOXO3* gene is associated with human longevity and aging phenotypes [33]. Mice carrying triple conditional deletions of *FOXO1*, *FOXO3a*, and *FOXO4* genes in the adult hematopoietic system exhibited myeloid lineage expansion, lymphoid developmental abnormalities, and a decreased long-term repopulation ability in vivo while increased ROS levels [30]. Another study from Dr. Tosho Suda’s group also demonstrated that *FOXO3a*-deleted HSCs can neither maintain quiescence nor support long-term reconstitution of hematopoiesis in vivo [34]. *FOXO3a* deficiency increased levels of ROS and downregulated several cyclin-dependent kinases inhibitors, resulting in the exit of HSCs from quiescence. This boosted sensitivity to cell-cycle-specific myelotoxic injury, and loss of self-renewal capacity during aging [34]. *FOXO3a* knockout HSCs also showed lower expression of mitochondrial *Superoxide dismutase 2* (*SOD2*) and *Catalase*, two FOXO targets involved in ROS detoxification [35,36]. Further, FOXO3 has been shown to be crucial for the regulation of mitochondrial respiration in HSCs, which, under disrupted conditions, generate more ROS [29]. This strengthens the hypothesis that *FOXO3a* deficiency causes HSCs cell cycle abnormalities via mitochondrial ROS dysregulation.

Mitochondrial ROS levels and the related signaling pathways, thus, represent a major player in regulating the long-term self-renewal, activation, proliferation, differentiation, and aging of HSCs. Similar roles could also be played by extrinsic factors and the surrounding microenvironment (see Box 1), which can have a direct impact on ROS levels and the signaling pathways regulating HSCs homeostasis [10].

ROS are produced as by-products of mitochondrial respiration; their production is increased, and they are accumulated when mitochondrial respiration is altered. The major ROS source is the mitochondrial electron transport Chain (ETC), which is widely targeted by mitochondrial DNA (mtDNA) mutations [37], as described in detail in the following dedicated paragraphs.

Box 1 **Bone Marrow Microenvironment.**

Upon aging, drastic changes to the bone marrow microenvironment may serve as an extrinsic factor that promotes HSC aging. These changes include higher levels of several niche-derived soluble factors, such as the pro-inflammatory CC-chemokine ligand 5 (CCL5), osteopontin, and CXCL12 [38,39,40], as well as niche anatomical remodeling [41,42]. Pro-inflammatory cytokines increase with age in the bone marrow microenvironment of both mice and humans, driving myeloid differentiation [43]. In aged-related myeloid malignancies, such as myeloid proliferative neoplasms and chronic myeloid leukemia, serum interleukine (IL)-1β and IL6 levels are elevated [44,45]. Further studies will clarify whether inflammation is the cause or the consequence of HSC aging. Anatomical and functional remodeling of the HSC niche accelerates myeloid-lineage cell expansion during aging. Maryanovich et al. demonstrated that bone marrow vasculature and its associated stromal cells are remodeled in elder mice, and that this is associated with progressive deterioration of the sympathetic nervous system (SNS). SNS neuropathy is an early driver of niche aging, and loss of SNS by surgical denervation or genetic deletion of neurotransmitter-targeting β3 adrenergic receptors induces the remodeling of the HSC niche and leads to premature aging-like changes in HSCs [46]. Another study also suggested that, during normal aging, increased β2 adrenergic receptors activity promotes IL6-dependent myeloid differentiation and subsequent premature HSC aging [47].

## 3. Mitochondrial Metabolism 

Despite the high preference of mitochondria for glycolysis, recent studies have also highlighted the importance of mitochondrial respiration to HSC for proliferation and maintenance [48,49,50]. We have recently shown that HSCs have a relatively high number of mitochondria, which are not completely inactive [51]. Indeed, mitochondrial membrane potential (ΔΨ_mt_) is high in HSCs, although ATP production or intracellular ROS levels are low [52,53]. The higher complex II: complex V ratio gives rise to high ΔΨ_mt_ in HSCs due to limited coupling of the electron transport chain (ETC), which supports the idea that mitochondrial complex II is pivotal for both HSC maintenance and the prevention of the aging process [53]. Indeed, inhibition of complex II reduces the in vitro colony-replating capacity of HSCs [53], and genetic mutation of *mev-1*, a subunit of the succinate dehydrogenase cytochrome b enzyme, which is a component of complex II, leads to oxygen hypersensitivity and premature aging of HSCs [54]. Studies of *C. elegans* uncovered that the mutated or silenced components of ETC or the ATP synthase can markedly extend [55,56,57] or reduce lifespan [58]. Although these varying experimental results must eventually be resolved, it is clear that imbalances in ETC activity are closely linked to the overall survival of the organism. Interestingly, a recent paper showed that ΔΨ_mt_ is a source of heterogeneity in old HSCs, with a prevalent fraction of low ΔΨ_mt_ in aged HSCs. Enhancement of ΔΨ_mt_ by mitoquinol (Mito-Q), a mitochondrial-targeted coenzyme-Q10 [59], successfully increased ΔΨ_mt_ of old HSCs and ameliorated or prevented onset of aging phenotypes [60].

HSCs are mainly dormant but can become highly active on demand, either to maintain hematopoietic homeostasis by replenishing matured/immature hematopoietic cells, or to respond to situations of emergency, such as infection or blood loss [61]. This shift requires a metabolic switch from glycolysis to mitochondrial oxidative phosphorylation, which is precisely regulated by various signaling pathways. The mammalian TOR (mTOR) pathway is a key regulator of cellular and mitochondrial metabolism. mTOR directly controls the mitochondrial oxidative function through a YY1–PGC-1α (peroxisome proliferator-activated receptor gamma coactivator 1-alpha) transcriptional complex [62]. Defects of *Tuberous sclerosis complex subunit 1* (*TSC1*), the major negative regulator for mTOR [63], lead to increased mitochondrion biogenesis and accumulation of ROS. Blockade of ROS activity in vivo restores these HSC defects, demonstrating that the TSC-mTOR pathway controls the quiescence and on-demand functions of HSCs by repressing ROS production [64,65].

HSCs exhibit low AKT/mTOR activity, but, upon stress, upregulation of this pathway drives dormant HSCs toward activation [64,66]. Interestingly, the dysregulation of AKT/mTOR signaling correlates with the aging process in HSCs [67]. Experimental evidence has shown that mTOR activation is involved in HSC aging, as well as that rapamycin treatment restores HSC potential and prolongs the lifespan of mice [68]. mTOR activity is higher in HSCs from elder mice than younger mice, and mTOR activation, through conditional deletion of *TSC1* in the HSCs of young mice, mimics the phenotype of HSCs from aged mice; similarly, in older mice, rapamycin restores the self-renewal capacity of HSCs and, importantly, correlates with increased life span [68].

*ASXL1* is frequently mutated in age-related clonal hematopoiesis. Its mutation activates the AKT/mTOR pathway, causing aberrant cell cycle progression in the HSC compartment and provoking HSC dysfunction. This is associated with mitochondrial activation, elevated ROS levels, and increased DNA damage, leading to age-associated phenotypes, such as myeloid-biased differentiation, hypocellular bone marrow, and increased frequency, of phenotypic LT-HSCs. Inhibition of the AKT/mTOR pathway can partially rescue these phenotypes, suggesting its involvement in the enhanced aging of the hematopoietic system [69].

A similar phenotype is observed in wild-type p53-induced phosphatase 1 (WIP1), which is highly expressed in HSCs but decreases with age. *WIP1*-deficient (*WIP1*^−/−^) mice exhibit multiple aging-like phenotypes in HSCs, including declines in reconstitution ability and HSC expansion. Mechanistically, their impaired regenerative capacity is due to a p53-mediated differentiation defect, whereas increasing numbers of *WIP1*^−/−^ HSCs are associated with mTOR-mediated cell cycle progression of HSCs [70]. Notably, experimental results have shown that aged HSCs have higher mTOR [71] activity levels, as well as that its inhibitor rapamycin can restore the self-renewal of aged HSCs, an effect which can be translated to human HSCs [72].

Recent advances have demonstrated that epigenetic, transcriptional, and post-transcriptional mechanisms also control the quiescence of HSCs, which are maintained in a paused state that allows for rapid activation [73]. Mitochondrial activity modifies the epigenetic state of cells affecting their aging process [74]. Citric acid, generated by the tricarboxylic acid (TCA) cycle in the mitochondria, modulates histone acetylation and gene expression through its conversion to acetyl-CoA [74]. Mitochondrial fatty acid oxidation (FAO) also generates acetyl-CoA for histone modification in HSCs [75]. 

Sirtuins are a family of protein deacetylases, which regulate the mitochondrial metabolic checkpoint in stem cells, and they are key regulators of stem cell aging [21,76]. SIRT3 plays a critical role in the mitochondria, where it deacetylates two critical lysine residues on SOD2 to promote the antioxidative activity. Brown and colleagues have demonstrated that SIRT3 is highly enriched in HSCs, as well as is suppressed with aging [77]. Although SIRT3 has no effect on HSCs maintenance or tissue homeostasis at a young age under homeostatic conditions, it is essential under stress or in old age. Indeed, SIRT3 loss induces HSC quiescence and compromises regenerative capacity in old mice [77].

SIRT1 is a key regulator of HSCs self-renewal and lineage specification under homeostasis. Interestingly, Ghaffari’s group has shown that loss of *SIRT1* causes anemia and myeloid expansion at the expense of the lymphoid compartment, overlapping features with aged HSCs. SIRT1 plays a role in HSCs homeostasis by targeting FOXO3, a longevity transcription factor and mitochondrial ROS regulator [78].

Another key regulator of metabolism is nicotinamide adenine dinucleotide (NAD^+^). Decreased levels of NAD^+^ are associated with cancer, metabolic disorders, and physiological and accelerated aging processes [79,80,81]. Supplementation of nicotinamide riboside (NR), a NAD^+^ precursor, significantly improved lifespan and health span in model of aged-related disease, such as ataxia–telangiectasia mutation (ATM), thanks to the improvement of both DNA damage repair and mitophagy [71,82]. Murine models of ATM loss show defects in DNA damage repair associated with mitochondrial dysfunction [83] and loss of hematopoietic stem cell (HSC) potential [23]. NR treatment caused significant alterations in lineage commitment of HSCs with enhanced lymphoid potential [84].

## 4. Mitochondrial Quality Control

Increased evidence indicates that mitochondrial integrity is disrupted during aging, and this contributes to the pathogenesis of age-related disorders in humans [3,85]. Mitochondria have evolved multiple mechanisms to guarantee mitochondrial quality. For instance, mitochondrial chaperones and proteases prevent the accumulation of misfolded and aggregated proteins by monitoring proteostasis through the mitochondrial unfolded protein stress response (mtUPR) [86], a mechanism that has been shown to be critical for longevity in mammals [87,88]. The mtUPR is a cellular protective program that ensures proteostasis in the mitochondria and is activated by mitochondrial protein folding stress [89]. mtUPR has recently emerged as a regulatory mechanism for adult stem cell maintenance [21,90,91,92]. This protective program is dysregulated during physiological aging, which contributes to the functional deterioration of stem cells, tissue degeneration, and shortened organismal lifespan [21,91]. SIRT7 is an NAD^+^-dependent H3K18Ac (acetylated lysine 18 of histone H3) deacetylase originally studied for its role in cancer cells [93]. Mohrin et al. have shown that *SIRT7* represses the expression of mitochondrial ribosomal proteins in order to regulate mtUPR and reduce mitochondrial protein folding stress [21]. *SIRT7* ablation leads to loss of quiescence and aging phenotypes in HSCs, including reduced regenerative capacity and the myeloid-biased differentiation [21].

Stem cells exhibit high levels of autophagy as part of their physiological state [94]. Autophagic activity is necessary for the self-renewal and differentiation capacities of stem cells, particularly HSCs [95]. Autophagy is closely linked with health and longevity, and impaired levels of autophagy in aged HSCs leads to the accumulation of mitochondria, which in turn induces metabolic stress [96,97]. Lysosomal sequestration of mitochondrial enhances the regenerative capacity of HSCs [98]. Overall, autophagy declines in aged stem cells, contributing to loss of quiescence, senescence, and, ultimately, degeneration [99].

Deregulation of other compensatory mitochondrial protective programs, such as mitophagy and mitochondrial dynamics, also affect stem cell maintenance highlighting the importance of mitochondrial integrity [95,96,97,100,101,102]. Indeed, mitochondria are dynamic organelles existing in large tubular and highly dynamic networks that constantly undergo fission and fusion processes, thereby leading to the dilution of non-functional mitochondria [103]. The effects of mitophagy and mitochondrial fusion/fission process on health and lifespan has been particularly demonstrated by using the model organisms *C. elegans* and D. melanogaster. For instance, the overexpression of the mitochondrial fission protein dynamin-related protein 1 (DRP1) increased the healthy lifespan in flies [104]. The importance of mitochondrial fission on drosophila lifespan was further demonstrated by the observation that lifespan extension caused by the overexpression of p62 was abrogated in DRP1 mutant flies [105]. Lifespan extension in flies was also observed after overexpression of mitophagy key proteins, such as PARKIN and PINK1 [106,107]. These findings are consistent with studies in *C. elegans*, where mitophagy has been shown to contribute to lifespan regulation [108,109].

## 5. Inflammation

The natural aging process is associated with activation of the innate immune system, which results in a low-grade chronic pro-inflammatory status, even in the absence of overt diseases [43,110]. Aged-related inflammation (inflammaging) is due to the systemic overabundance of pro-inflammatory cytokines, such as IL-1, tumor necrosis factor (TNF), and IL-6 [111,112]. Several studies focused on HSCs have shown that inflammatory signaling induces the differentiation of the myeloid progenitor cells required to withstand harmful stimuli [112,113,114]. Specifically, IFN-α and IFN-γ activate HSCs entry into the cell cycle and boost the myeloid-biased differentiation of HSCs [115,116], as well as IL-1β, which promotes myeloid lineage biased differentiation of HSCs [117].

Notably, this cytokine network, termed the senescence-associated secretory phenotype (SASP) [118], may be initiated by senescent cells producing IL-1α in the bone marrow microenvironment (Box 1) [119]. HSCs do not solely show mitochondrial defects; for example, CD4^+^ T cells from elderly people display an elevated number of dysfunctional mitochondria engulfed into autophagosomes compared to cells from young people, suggesting the presence of a defective mitochondrial turnover. These defective mitochondria may be the source of inflammatory stimuli and contribute to the impairment of immune defenses in the elderly [120,121].

Aged HSCs exhibit increased NF-κB activity mediated by RAD21/cohesion, which enhances sensitivity to inflammatory stimuli, higher production of IL-6, and myeloid-biased hematopoietic differentiation [122]. Recently, the Wang group has shown that systemic level of TNF-α, a well-known biomarker of inflammation, increases with age and induces the expression of IL27Ra in HSCs via ERK-ETS1 signaling [123]. The chronic inflammatory process associated with aging leads to dysfunctional differentiation of stem cells, loss of self-renewal capacity, and further promotion of HSC aging [124].

The relevance of mitochondria to the pro-inflammatory response has been largely studied as a source of damage-associated molecular patterns (DAMPs) during cell death [125]. Mitochondrial DAMPs include mtDNA, cytochrome c, ROS, and ATP, which, when released in the cytosol, are recognized by the cell as a red flag of danger and trigger apoptosis or necrosis [125]. Besides pro-apoptotic signals, mitochondria DAMPs are also potent immunostimulators. The exposure to mtDNA triggers a variety of innate immune responses due to its bacterial origin [126], and the modulation of ROS signaling causes the activation of the principal component of innate immunity, the NOD-, LRR-, and pyrin domain-containing 3 (NLRP3) inflammasome [127,128].

The use of specific mitochondria ROS scavenger, the mito-TEMPO, inhibit the NLRP3 inflammasome activation, reducing the up-regulation of IL-1β and IL-18 induced by lipopolysaccharide (LPS) [127,129]. Luo et al. recently demonstrated that mitochondrial stress activates the NLRP3 inflammasome in HSCs as part of the key role played by mitochondria in the inflammation process during aging [130]. ROS are a metabolic danger signal and activate an innate immune sensor, the NLRP3 inflammasome. Once triggered, the NLRP3 inflammasome induces pro-inflammatory cytokine secretion and/or caspase 1-dependent cell death [131]. *NLRP3* is highly expressed and studied in myeloid cells; however, it is also expressed and functions in HSCs [130]. NLRP3 is a substrate of SIRT2, which inhibits NLRP3 activity through deacetylation [132]. The reduced expression of *SIRT2* in aged HSCs enhances the activity of the NLRP3 inflammasome, which increases susceptibility to mitochondrial stress-induced stem cell deterioration. Interestingly, the overexpression of *SIRT3* or *SIRT7* (described above) reduces caspase 1 activity and improves the function of aged HSCs [130], tightening the link between mitochondria and inflammation. 

Finally, patients with high levels of circulating mtDNA (described in the next section) show higher concentrations of IL-6, TNF-a, RANTES, and IL-1 [133].

## 6. Mitochondrial DNA Mutations

The human mtDNA genome encodes 13 genes, 22 tRNAs, and 2 rRNAs [134]. These 13 genes encode for core subunits of the electron transport chain (ETC) complexes and the ATP synthase. During aging, mtDNA accumulates mutation, causing dysfunction of mitochondria and the respiratory chain [135]. Several papers proposed that mtDNA mutations play a key role in aging [3,136,137]. mtDNA has a much higher mutation rate than nuclear DNA [138,139]. Indeed, the mtDNA spatial proximity to the site of ETC-mediated ROS production make it particularly susceptible to ROS damage. In addition, differently from the nuclear DNA, mtDNA cannot be organized in highly compacted structures by histones, enhancing the probability of coping errors introduced during replication [140]. Interestingly, it has been reported that the aged population has a higher mtDNA copy number in blood [141,142] and exhibits mtDNA heteroplasmy, i.e., the presence of more than one type of organelle genome [143,144]. Of note, individual mtDNA mutations were found in centenarians [145]. One example is represented by A5178C mutation in MT-ND2 gene, which encodes the NADH dehydrogenase 2, a subunit of ETC complex I. This mutation confers protection on mitochondria against oxidative damage contribute to longevity [146]. 

Conplastic mice strains are a suitable model system for the study of specific mtDNA variations and their influence on ROS and ATP levels upon aging [147]. In this paper, conplastic mouse strains C57BL/6Ntac-mt**^AKR/J^** (mtAKR), C57BL/6Ntac-mt^129S1SvlmJ^ (mt129S1), C57BL/6Ntac-mt^NOD/LTJ^ (mtNOD), and the background strain C57BL/6Ntac (B6Ntac) were used to investigated their hematopoietic changes during aging. The three mouse strains harbor specific polymorphisms in the mtDNA affecting complex I, III, and IV of respiratory chain [148]. The presence of mtDNA polymorphisms in these subunits of the respiratory chain decreased intracellular ROS levels and lymphocyte counts during aging [148].

Although somatic mtDNA mutations accumulate in multiple tissues with age [136], its causal role in tissue aging remains to be clarified [149]. The direct effects of mtDNA mutations have been studied through the analysis of a mouse model carrying a proofreading-defective mitochondrial DNA polymerase (POLGA^D257A^) [150]. The consequent accumulation of mtDNA mutations led to premature aging of the mice, which showed hair loss, weight loss, osteoporosis, anemia, and myeloid lineage skewing with lymphopenia [150,151,152,153,154]. Accumulating mtDNA point mutations destabilized ETC complexes I, III, and IV, leading to respiratory chain deficiency [155]. The POLGA^D257A^ did not show an increase in ROS production [156], but an antioxidant treatment has separately been shown to rescue erythroid differentiation in embryos [151,152]. Chen et al. showed that POLGA^D257A^ mutant animals develop an age-dependent, macrocytic anemia with abnormal erythroid maturation and megaloblastic changes, as well as profound defects, in lymphopoiesis. These abnormalities phenocopy patients with myelodysplastic syndrome (MDS) and refractory anemia, suggesting that abnormalities of mitochondrial function can be involved in the pathogenesis of the diseases [157].

In humans, *POLG* (*DNA Polymerase Subunit Gamma*) mutations were not linked to symptoms of premature aging, even though they are one of the most frequent causes of mitochondrial disease [158]. Mice carrying a defect in mitochondrial genome maintenance exonuclease 1 (*MGME1*) showed mtDNA replication defects and developed a severe multisystemic mitochondrial disorder [159] without signs of premature aging [160]. All things considered, mitochondrial dysfunctions caused by accumulating mtDNA mutations has been shown to cause multiple hematopoietic defects that are typically seen in the elderly, but mtDNA mutations alone may not be responsible for the phenotype associated with aging HSCs [161,162,163].

Dr. Sankaran’s group has recently presented a new fascinating tool for the studying of mtDNA mutation. They developed a high-throughput platform for measuring mtDNA mutation heteroplasmy, along with accessible chromatin states, in thousands of single cells [164,165]. Performing clonal tracing in human hematopoiesis in vivo has the potential to resolve the clonal heterogeneity within malignancies and the aging process. 

## 7. Conclusions and Future Perspective

With a better understanding of the mechanisms of HSC aging, researchers will be able to explore new opportunities to prevent, delay, or even reverse aspects of this process. In this review, we summarize accumulating evidence that support the concept that mitochondrial stress is one of the main drivers of stem cell deterioration with age. Targeting mitochondrial protective pathways could, therefore, allow us to protect stem cells from aging, with important therapeutic implications.

Since the studies of HSC aging have been performed, for the most part, in mouse models, it is crucial to determine whether these findings can be translated to humans. Increasing evidence has revealed a phenomenon termed age-related clonal hematopoiesis (ARCH), describing a clonal expansion of blood cells derived from mutated HSCs in aged humans [166]. Single-cell sequencing of sorted cell populations is able to identify even subclonal mutation inside the stem cell pool, revealing the high complexity of this process [167]. It would be of great interest to determine whether the mitochondria plays a role in the development of human clonal hematopoiesis.

Finally, a better understanding of the molecular properties of the clonal subclass of myeloid-biased HSCs may provide insights into the onset of clinically age-dependent hematological disorders/malignancies derived from stem cells. ARCH can be a precursor to MDS, and MDS is associated with age with a median age at diagnosis of 65–70 years. AML is an aggressive hematological disorder mainly affecting people of advancing age, and 30% of patients with AML are 75 years or older [168]. Notably, we recently revealed a mitochondrial ROS senescence pathway triggered by Nucleophosmin 1 mutant (NPM1c), PML, and TP53, which plays a crucial role in actinomycin D-based therapies in AML [169]. This supports the idea of mitochondria as potential therapeutic target.
